# Metabolome analysis reveals a diversity of cancer tissues in advanced epithelial ovarian cancer

**DOI:** 10.1186/s12935-021-02014-7

**Published:** 2021-06-16

**Authors:** Kosuke Yoshida, Nobuhisa Yoshikawa, Kazuhisa Kitami, Satoshi Tamauchi, Yoshiki Ikeda, Akira Yokoi, Kimihiro Nishino, Kaoru Niimi, Hiroaki Kajiyama

**Affiliations:** 1 Department of Obstetrics and Gynecology, Nagoya University Graduate School of Medicine, Tsuruma-cho 65, Showa-ku, Nagoya, 466-8550 Japan; 2 Institute for Advanced Research, Nagoya University, Nagoya, Japan

**Keywords:** Metabolome, Epithelial ovarian cancer, Omental metastasis

## Abstract

**Background:**

Epithelial ovarian cancer remains one of the leading causes of cancer deaths among women worldwide, and advanced epithelial ovarian cancer frequently metastasizes to the omentum. The characteristics of metastatic cancer may differ from those of primary ovarian cancer and reflect the unique omental microenvironment. This study investigated metabolomic differences in epithelial ovarian cancers.

**Methods:**

Patients with advanced epithelial ovarian cancer were eligible for this study. Five patients underwent surgery and resection of paired primary ovarian and omental metastatic cancer at Nagoya University. Metabolome analysis was performed in these paired cancer and metastatic cancer tissues through a facility service (C-SCOPE) at Human Metabolome Technologies, Inc. The concentrations of 116 compounds were measured by CE-TOFMS and CE-QqQMS, and 30 metabolic parameters were calculated. For statistical analyses, Welch’s *t*-test was used for comparisons between two independent groups.

**Results:**

Metabolite profiles were all different, which reflects diversity among these cancer tissues. Of the measured compounds, urea was the only metabolite that was significantly decreased in omental metastatic cancers compared with the primary cancers (p = 0.031). Moreover, in omental metastatic cancers, the pentose phosphate pathway was more dominant than glycolysis. Furthermore, in some cases, lactic acids in omental metastatic cancers were markedly decreased compared with primary cancers. With regard to histological subtype, the total levels of amino acids, especially the percentage of glutamine, were significantly enriched in serous carcinomas compared with nonserous carcinomas (p = 0.004 and p = 0.001). Moreover, the reduced forms of glutathione and polyamines were also more abundant in serous carcinomas than in nonserous carcinomas (p = 0.025 and 0.048).

**Conclusions:**

The metabolite profiles differed depending on tumor location and histological subtype. Metabolome analysis may be a useful tool for identifying cancer diagnostic and prognostic markers.

**Supplementary Information:**

The online version contains supplementary material available at 10.1186/s12935-021-02014-7.

## Background

Epithelial ovarian cancer (EOC) is one of the leading causes of cancer-related deaths among women worldwide with an estimated 184,799 deaths in 2018 [1]. EOC comprises four major histological subtypes: high-grade serous ovarian carcinoma (HGSOC), clear cell carcinoma (CCC), endometrioid carcinoma (EC), and mucinous carcinoma, and the clinical and molecular biological features are different for each histological subtype [2–4]. The tumor microenvironment plays a critical role in cancer progression, and EOC typically spreads to the peritoneal cavity and frequently metastasizes to the omentum, which is characterized by an adipose-rich environment [4–8]. Several studies have indicated that adipocytes in the omentum contribute to cancer development by producing cytokines and chemokines [8–10]. This implies that the molecular biological characteristics of the cancer cells may change, thus reflecting their unique microenvironment. Therefore, understanding the differences is essential for elucidating EOC pathology.

Since cancer cell-specific metabolism, which is known as the Warburg effect, was discovered, energy metabolism in cancer tissue has been considered to be important [11–14]. Metabolome analysis represents a robust method that is used to identify and quantitate the levels of small molecules related to cellular metabolic activity, such as sugars, amino acids, and other organic molecules [15]. In molecular biology, the gene expression level is usually regarded as a surrogate for predicting gene function. However, in the case of enzymatic reactions that are part of a metabolic pathway, the enzyme expression level and its function are not necessarily correlated [16, 17]. Therefore, metabolome analysis is suitable for evaluating the functional differences in the microenvironment because it enables analysis of the metabolic changes that reflect enzyme function. Several studies have indicated that serum and tissue metabolites may be potential diagnostic and prognostic biomarkers of EOC [18–21]. However, metabolomic differences based on the spatial diversity of the tumor microenvironment remain undefined.

In this study, we investigated the metabolic changes in primary ovarian cancer (PC) and omental metastatic cancer (OMC) and the metabolic differences associated with the various histological subtypes of EOC.

## Methods

### Patients and samples

Archival fresh frozen EOC tissue samples were collected from five patients who underwent simultaneous PC and OMC tissue resection at the Nagoya University Hospital (Aichi, Japan) since 2018. Patient characteristics are provided in Table 1. Histologically, Cases 1 and 2 were HGSOC, Cases 3 and 4 were CCC, and Case 5 was EC. Case 3 underwent interval debulking surgery after eight courses of carboplatin and paclitaxel combination chemotherapy, whereas the remaining four patients underwent primary debulking surgery. The study protocol was approved by the Ethics Committee of our institution (approval No. 2017-0497), and written informed consent was obtained from each patient. Immediately following resection, cancer tissues were macroscopically sectioned into small pieces and placed into 1.5-mL tubes. They were immediately frozen in liquid nitrogen and stored at − 80 ℃. Approximately 40–50 mg of tissues from each case was used for the metabolome analysis.

**Table 1:** Patients’ characteristics

No	Age	Stage	Histology	NAC
1	71	IIIC	HGSOC	No
2	57	IIIC	HGSOC	No
3	50	IIIC	CCC	Yes
4	67	IIIC	CCC	No
5	63	IVB	EC	No

*HGSOC* high-grade serous ovarian carcinoma, *CCC* clear cell carcinoma, *EC* endometrioid carcinoma, *NAC* neoadjuvant chemotherapy

### Metabolite measurements

Metabolome analysis was performed using a facility service (C-SCOPE) at Human Metabolome Technologies, Inc. (Yamagata, Japan). Briefly, the tissues were homogenized in a 50% acetonitrile aqueous solution. The samples were then centrifuged at 2,300 × g at 4˚C for 5 min and the upper aqueous layer was centrifugally filtered through a 5 kDa cutoff filter at 9,100 × g for 120 min at 4˚C. The filtrates were resuspended in 50 μl of Milli-Q water and the metabolome analysis was performed using CE-TOFMS and CE-QqQMS. The data were analyzed by MasterHands ver.2.17.1.11 (Keio University, Tsuruoka, Japan) and MassHunter Quantitative Analysis B.06.00 (Agilent Technologies, Santa Clara, CA), and the peak area of each metabolite was calculated. The peak area was then normalized to an internal standard, and the metabolite concentrations were calculated using standard curves. Overall, 116 compounds were measured and 30 metabolic parameters were calculated.

### Data analysis and statistical analysis

RStudio (RStudio, Boston, MA) and R software (ver. 3.5.0) were used for the analysis. For heatmap and principal component analyses, values below the detection sensitivity were designated as zero, and the measurements of substances for which the concentration was zero were excluded from all samples. The data was converted to base 10 logarithms and z-scores. The heatmap.2 function of the gplots package (ver. 3.0.1) was used to generate a heatmap. The distance was calculated as “1–Spearman correlation coefficient” and the “ward.D2” clustering method was used. To calculate and visualize the principal component analysis, the prcomp and plot3d functions of the rgl package (ver. 0.99.16) were used. For statistical analyses, Welch’s *t*-test was used for comparisons between PC and OMC. In addition, metabolite changes between HGSOC and non-HGSOC were compared using Welch’s *t*-test. P-values less than 0.05 was considered statistically significant.

## Results

**Fig. 1:**
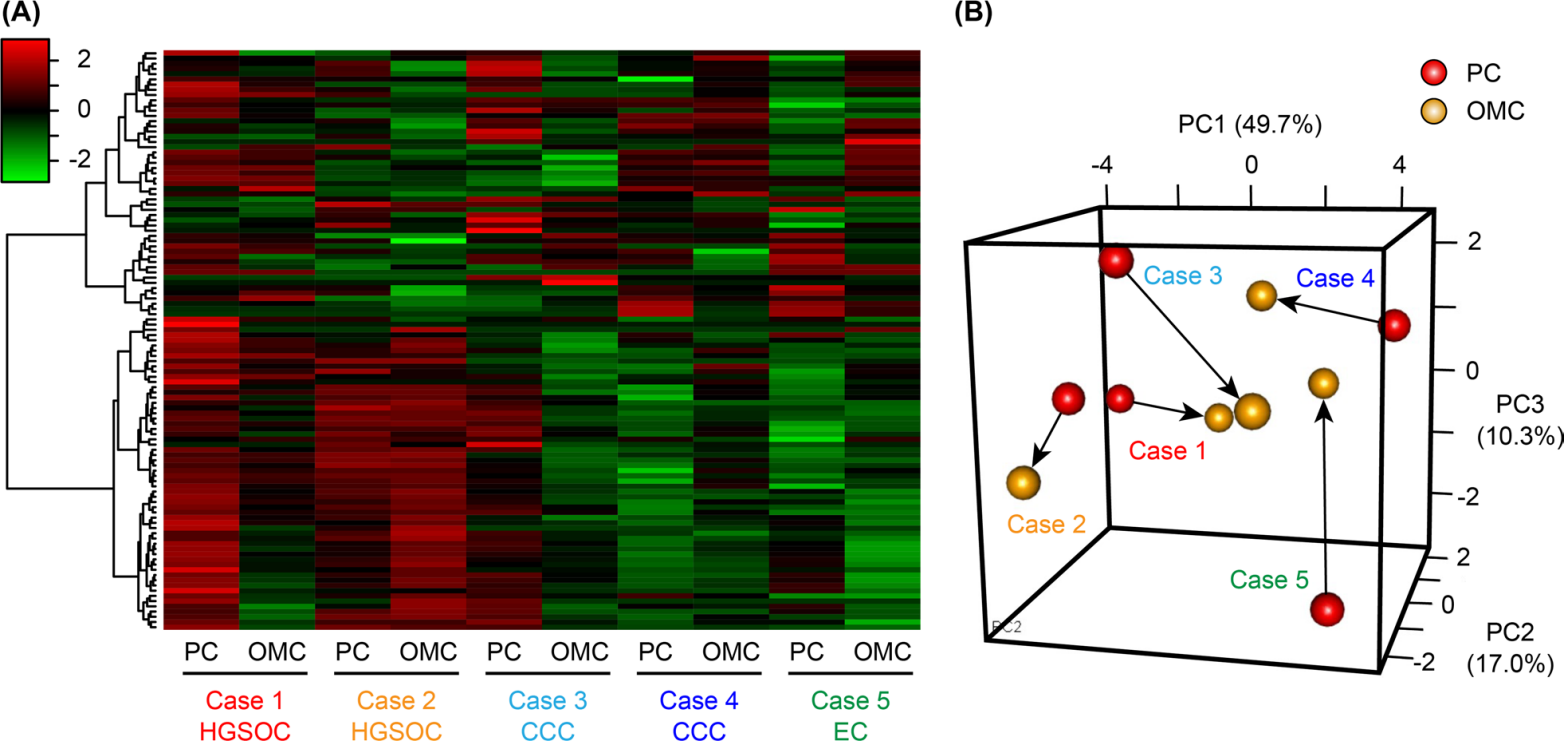
Metabolome analysis of primary cancer (PC) and omental metastatic cancer (OMC) tissues. **A** Heatmap analysis showing metabolite profiles of each sample. Cases 1 and 2 were high-grade serous ovarian carcinoma (HGSOC), Cases 3 and 4 were clear cell carcinoma (CCC), and Case 5 was endometrioid carcinoma (EC). **B** Principal component analysis of the metabolome analysis. Red and yellow indicate PC and OMC, respectively, and the same cases are connected by arrows

We performed a metabolome analysis using 10 fresh-frozen EOC tissues from five patients in which 116 compounds were measured (Additional file 1). A heatmap analysis revealed that metabolites from the paired PC and OMC tissues were similar and that metabolites in HGSOC subtype (Cases 1 and 2) were quite different from those in the CCC and EC subtypes (Cases 3–5, Fig. 1A). However, no significant differences were observed in the metabolite profiles between the PC and OMC groups. Likewise, the principal component analysis revealed a diversity in the cancer tissues based on histological subtypes and individual differences (Fig. 1B).

### The urea cycle

**Fig. 2:**
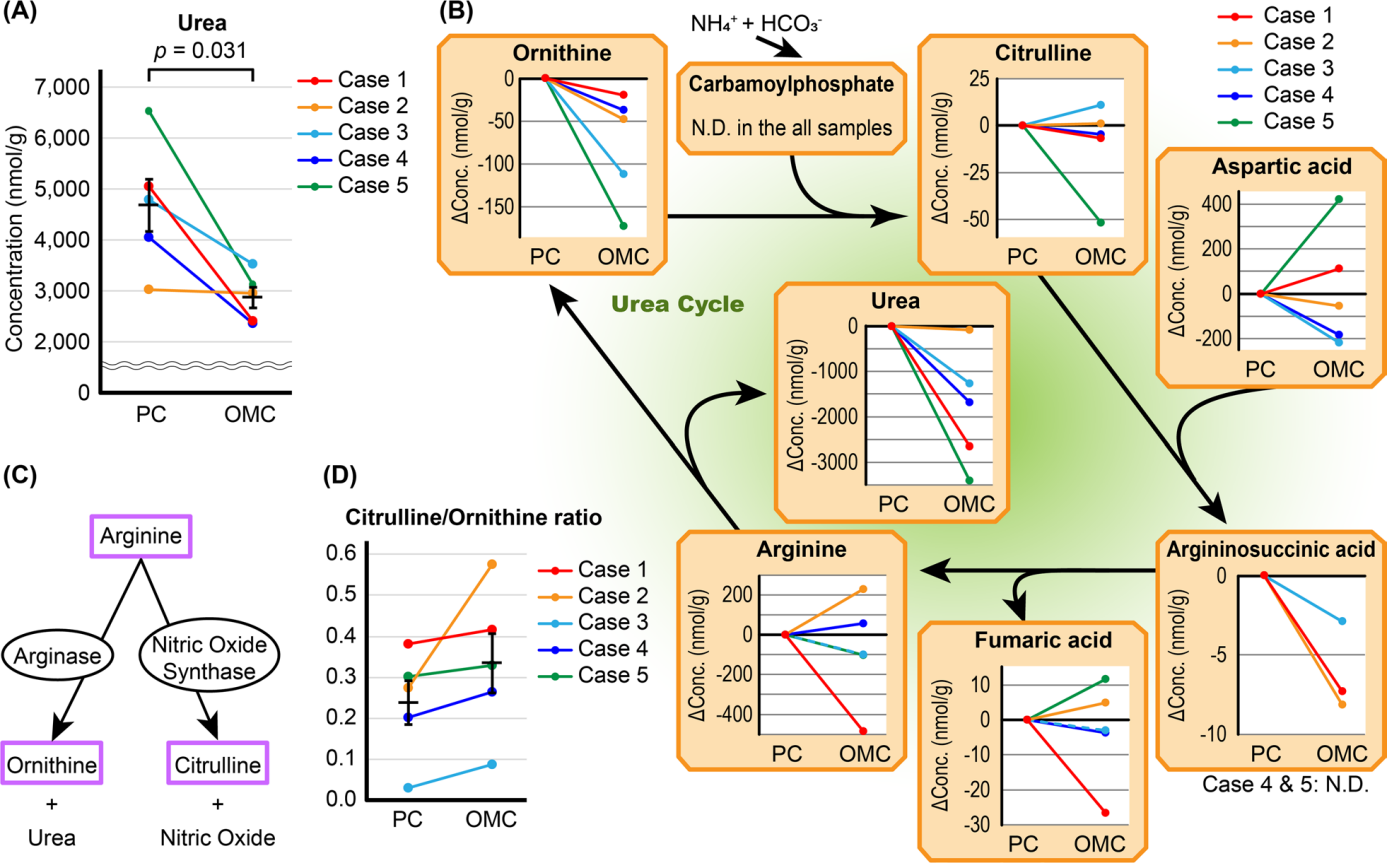
Metabolomic profiles related to the urea cycle. **A** Absolute urea concentration in primary cancer (PC) and omental metastatic cancer (OMC). The urea concentration was compared in PC and OMC by Welch’s *t*-test. **B** Metabolite changes related to the urea cycle. Changes in the concentration of each metabolite in OMC are shown relative to the corresponding PC. N.D., not detected. **C** Schema of two metabolic pathways of arginine. **D** Changes in the citrulline/ornithine ratio between PC and OMC. Error bars represent standard errors of the mean

We investigated the detailed differences in multiple metabolic pathways between the PC and OMC groups. The concentrations of most of the compounds were not significantly different between the two groups (Additional file 1); however, the urea concentration in the OMC group was significantly decreased compared with the PC group (p = 0.031). Notably, urea was decreased by approximately half in Cases 1, 4, and 5 (Fig. 2A). Therefore, other compounds in the urea cycle were subsequently evaluated. The concentration of ornithine in the OMC group was also decreased in all cases as both urea and ornithine are derived from arginine (Fig. 2B). In addition to arginase, nitric oxide synthase (NOS) is a metabolic enzyme of arginine, and the citrulline/ornithine ratio is considered to be an index of the enzyme activities of arginase and NOS (Fig. 2C) [22]. The citrulline/ornithine ratio was elevated in all cases in the OMC group, although citrulline alone was not significantly altered (Fig. 2B, 2D). Therefore, NOS activity was increased in the OMC group, but its levels were not significantly different from the other groups (p = 0.352, Additional file 2). No significant differences were observed in other compounds (Fig. 2B).

### Glycolysis and the pentose phosphate pathway (PPP)

**Fig. 3:**
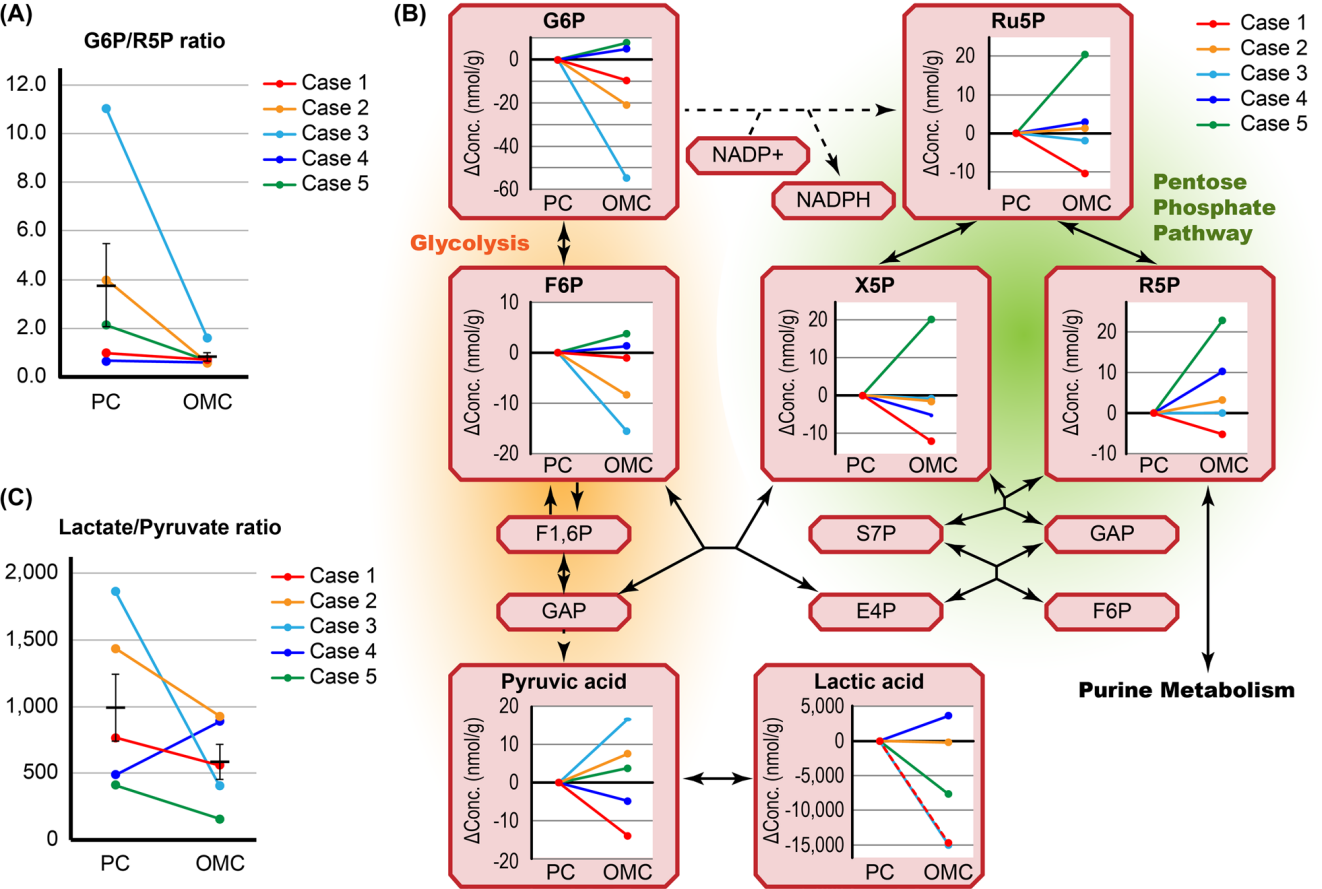
Metabolomic profiles related to glycolysis and the pentose phosphate pathway. **A** Changes in the glucose-6-phosphate (G6P)/ribose-5-phosphate (R5P) ratio between primary cancer (PC) and omental metastatic cancer (OMC). **B** Metabolite changes related to glycolysis and the PPP. Changes in the concentration of each metabolite in OMC are shown relative to the corresponding PC. **C** Changes in the lactate/pyruvate ratio between PC and OMC. Error bars represent standard errors of the mean. F6P, fructose 6-phosphate; F1,6P, fructose 1,6-bisphosphate; GAP, glyceraldehyde-3-phosphate; Ru5P, ribulose 5-phosphate; X5P, D-xylulose 5-phosphate; S7P, sedoheptulose 7-phosphate; E4P, erythrose 4-phosphate

Next, we focused on glycometabolism. The glucose-6-phosphate (G6P)/ribose-5-phosphate (R5P) ratio was decreased in the OMC group compared with the paired PC group, but the difference was not significant (p = 0.204, Fig. 3A and Additional file 2). G6P was somewhat decreased in the OMC group, especially in Case 3, whereas R5P was increased in the OMC group, particularly in Case 5 (Fig. 3B). This suggested that the PPP was dominant in the OMC group. However, the concentrations of NADP + and NADPH were almost identical in all the samples (Additional file 1).

Pyruvic acid is produced as a product of glycolysis and is metabolized to lactic acid and acetyl-CoA. Lactic acid was remarkably decreased in the OMC group in Cases 1, 3, and 5 (Fig. 3B and Additional file 1). Moreover, the lactate/pyruvate ratio was decreased in the OMC group compared with the paired PC group in all cases except Case 4 (Fig. 3C). However, due to individual differences, these differences were not statistically significant (p = 0.248). This suggested that the oxygen environment in OMC is different than that in PC.

### Amino acid composition

**Fig. 4:**
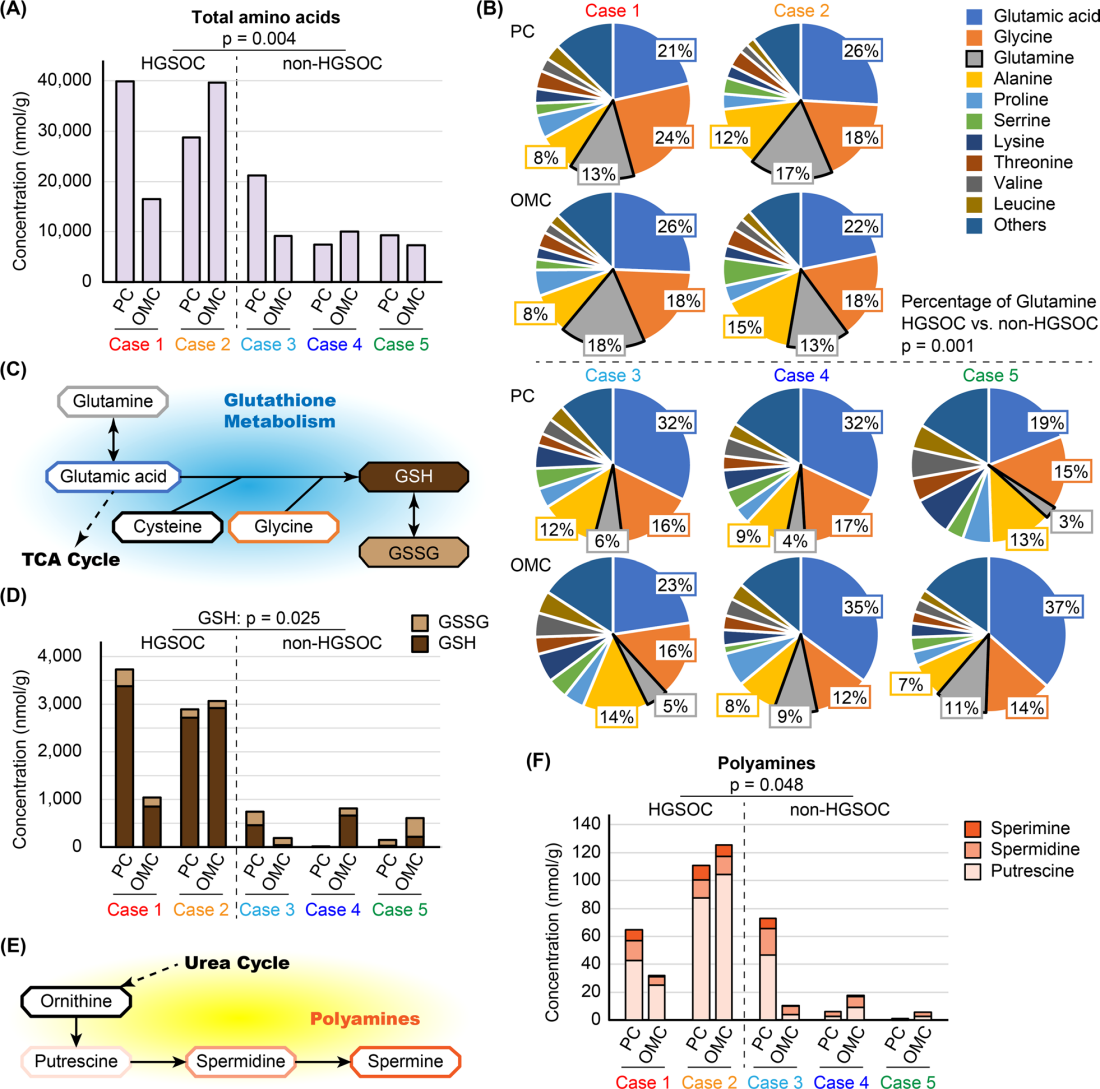
Metabolite differences based on histological subtype. **A** Absolute total amino acid concentration. Total amino acid concentration in high-grade serous ovarian carcinoma (HGSOC) and non-HGSOC was compared by Welch’s *t*-test, regardless of whether the sample was primary cancer (PC) or omental metastatic cancer (OMC). **B** The proportions of amino acids. The top 10 amino acids are shown, and the percentage of glutamine in HGSOC and non-HGSOC was compared by Welch’s *t*-test. **C** Schema of glutathione metabolism. Glutathione has two forms: a reduced form (GSH) and an oxidized disulfide form (GSSG). **D** Absolute GSH and GSSG concentrations. The GSH concentration was compared in HGSOC and non-HGSOC by Welch’s *t*-test. **E** Schema of polyamine metabolism. **F** Absolute polyamine concentration. The concentration of polyamines was compared in HGSOC and non-HGSOC by Welch’s *t*-test

In addition to the PC and OMC groups, we evaluated metabolite differences according to histological subtype. When HGSOC (Cases 1 and 2) was compared with non-HGSOC (Cases 3–5), HGSOC samples contained significantly more amino acids (p = 0.004, Fig. 4A). In all samples, glutamic acid was the major component, whereas glycine and alanine were also abundant (Fig. 4B). Notably, the proportion of glutamine was significantly higher in the HGSOC than in non-HGSOC samples (p = 0.001, Fig. 4B). As previously described, HGSOC samples contained more total amino acids, and thus, the absolute concentration of glutamine was also significantly higher (HGSOC vs. non-HGSOC, p = 0.004). Therefore, glutathione metabolism-related amino acids were enriched in HGSOC compared with non-HGSOC. (Fig. 4C). Glutathione has two forms; a reduced form (GSH) and an oxidized disulfide form (GSSG). GSH is converted into GSSG during oxidative stress [23]. The samples HGSOC contained significantly more GSH than the non-HGSOC (p = 0.025), whereas no differences in GSSG were observed (Fig. 4D). Ornithine, a metabolite of the urea cycle, is metabolized to polyamines including putrescine, spermidine, and spermine (Fig. 4E). The sum of these three polyamine forms was significantly higher in HGSOC samples than in non-HGSOC samples (p = 0.048, Fig. 4F).

## Discussion

Metabolism represents the set of life-sustaining chemical reactions in cells and is essential for cancer progression. In general, gene expression is regulated by various factors, such as microRNAs and 14–3-3 proteins [24–26]. Therefore, enzyme activity cannot be determined from the gene expression level alone, and analysis of metabolites is required [15, 17].

First, we showed that the urea concentration was significantly decreased in the OMC group compared with the PC group and that alterations in the urea cycle were observed. The urea cycle is an essential pathway for the disposal of ammonia, and thus, the relationship between alterations in the urea cycle and cancer progression has been presented [27, 28]. Our results indicated that the citrulline/ornithine ratio was increased in OMC and is considered to reflect decreased arginase activity and increased NOS activity. Consequently, NO was also considered to be increased. The function of NO is somewhat ambivalent, but NO primarily promotes cancer-related events, such as angiogenesis, apoptosis, and cell cycle [29]. Unfortunately, in this analysis, NO was not a target compound and further studies are needed to evaluate urea cycle dysfunction and NO during EOC progression.

Next, we focused on glycometabolism, which is essential for cell survival and unique to cancer tissues according to the Warburg effect [11–14]. In addition to glycolysis, the PPP is also a major pathway for glucose catabolism and plays an important role in cancer cell survival and growth [14, 30–32]. The PPP is divided into two biochemical branches known as oxidative and non-oxidative PPP. In the first, the chemical reaction is irreversible and two molecules of NADPH are generated from one molecule of glucose, whereas, in the other phase, which is reversible, the five-carbon sugar is generated from a six-carbon glucose molecule. Therefore, depending on which phase of the PPP is activated, the production of either NADPH or R5P is regulated [30–32]. In our results, the low G6P/R5P ratio in OMC indicated the predominance of the PPP. However, NADPH concentrations were similar in all the samples. Therefore, NAPDH consumption may have been increased in OMC due to oxidative stress. Alternatively, the OMC cells may produce more R5P than NADPH by activating the non-oxidative phase of the PPP. In addition, enhanced PPP metabolism might be due to the increased proportion of cancer stem cells in OMC tissues [30, 33]. Next, we evaluated glycolysis as the Warburg effect predicts that cancer cells use glycolysis even under aerobic conditions [11, 14]. We discovered that the lactate/pyruvate ratio was decreased in OMC, which suggested that the oxygen environment is different at metastatic sites and that cancer cells may change energy metabolism process based on their environment. Alterations of glycolysis between PC and OMC were also previously reported, although those detailed metabolic changes were not consistent with our results [34]. Therefore, the heterogeneity of cancer cells and individual differences may be significant and further larger-scale studies are needed.

We demonstrated that HGSOCs contained significantly more glutamine and other amino acids than non-serous carcinomas. Glutamine was the most prominent amino acid, and since the 1950s, it has been reported that proliferating cells require glutamine [14, 35]. In addition, highly proliferative cells produce high levels of reactive oxygen species (ROS) [36]. NADPH and GSH play important roles in the maintenance of intracellular redox balance, and elevated GSH levels are observed in various tumor types [23, 37]. We observed high glutamine and GSH levels in HGSOC, which may reflect a high proliferative potential. Moreover, GSH is known to mediate resistance to platinum analogs through several mechanisms including increased DNA repair and the inhibition of apoptosis [37, 38]. In addition, polyamines were also abundant in HGSOC. Polyamines are involved in many fundamental processes of cell growth and survival, and polyamine metabolism is associated with cancer-driving pathways [39]. In addition, a previous study reported that cyclin E1-driven EOC was associated with activated polyamine synthesis and decreased cancer immunity [40]. Therefore, GSH and polyamines are potential therapeutic targets in EOC, especially HGSOC.

This metabolome-based study has several limitations. First, we included only five cases of EOCs. Hence, although it is difficult to make any significant conclusion, the results presented in this study are very interesting. Therefore, further evaluation is essential to elucidate the metabolic changes in EOCs. Second, detailed molecular biological changes such as genetic mutations, mRNA and protein expression, and enzyme activities were not evaluated. Third, a lipidomics analysis was not performed, although this would be an interesting approach because the omentum is characterized by an adipose-rich microenvironment. However, lipid metabolites are not measurable with of C-SCOPE. Therefore, any metabolomic changes related to those molecules are worth evaluating in the future.

## Conclusion

Metabolome analysis revealed different metabolite profiles between PC and OMC, particularly in the urea cycle, glycolysis, and the pentose phosphate pathway. Moreover, HGSOC samples contained more amino acids and GSH than non-serous carcinoma samples. These differences may reflect the diversity of biological processes in cancer cells. Metabolome analysis is expected to be a powerful tool in cancer biology.

## Supplementary Information

**Additional file 1: Table S1.** Absolute quantitative values of 116 compounds.

**Additional file 2: Table S2.** Metabolic parameters based on the 116 compounds.

## Data Availability

All data generated or analyzed during this study are included in this published article and its Additional files.

## Abbreviations

EOC: Epithelial ovarian cancer
HGSOC: High-grade serous ovarian carcinoma
CCC: Clear cell carcinoma
EC: Endometrioid carcinoma
PC: Primary ovarian cancer
OMC: Omental metastatic cancer
NOS: Nitric oxide synthase
PPP: Pentose phosphate pathway
G6P: Glucose-6-phosphate
R5P: Ribose-5-phosphate
GSH: Reduced glutathione
GSSG: Oxidized glutathione
ROS: Reactive oxygen species
